# *N*,*N*′-Bis(4-pyridylmethyl­ene)octane-1,8-diamine

**DOI:** 10.1107/S1600536809024623

**Published:** 2009-07-01

**Authors:** Goutam Kumar Patra, Anindita Mukherjee, Partha Mitra, Seik Weng Ng

**Affiliations:** a Department of Chemistry, Vijaygarh Jyotish Ray College, 8/2 Vijaygarh, Jadavpur, Kolkata 700 032, India; b Department of Chemistry, University of Malaya, 50603 Kuala Lumpur, Malaysia

## Abstract

The complete molecule of the title compound, C_20_H_26_N_4_, is generated by a crystallographic centre of inversion and the central eight-carbon chain adopts a fully extended conformation.  In the crystal, the molecules pack in layers parallel to (010).

## Related literature

There are only few crystallographic reports of Schiff bases derived from 1,2-octa­nediamine; for details, see: Glidewell *et al.* (2005 ▶); Nathan *et al.* (2003 ▶); Viossat *et al.* (1997 ▶); Yamashita *et al.* (2003 ▶).
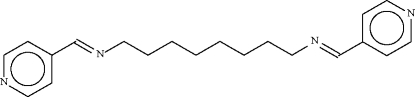


## Experimental

### 

#### Crystal data

C_20_H_26_N_4_
*M* *_r_* = 322.45
Monoclinic, 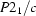

*a* = 11.6285 (4) Å
*b* = 9.3821 (3) Å
*c* = 8.8302 (3) Å
β = 111.143 (2)°
*V* = 898.52 (5) Å^3^
*Z* = 2
Mo *K*α radiation
μ = 0.07 mm^−1^
*T* = 140 K
0.40 × 0.20 × 0.02 mm

#### Data collection

Bruker SMART APEX area-detector diffractometer
Absorption correction: none
6110 measured reflections
2065 independent reflections
1588 reflections with *I* > 2σ(*I*)
*R*_int_ = 0.023

#### Refinement

*R*[*F* ^2^ > 2σ(*F* ^2^)] = 0.048
*wR*(*F* ^2^) = 0.138
*S* = 1.02
2065 reflections
109 parameters
H-atom parameters constrained
Δρ_max_ = 0.31 e Å^−3^
Δρ_min_ = −0.23 e Å^−3^

### 

Data collection: *APEX2* (Bruker, 2008 ▶); cell refinement: *SAINT* (Bruker, 2008 ▶); data reduction: *SAINT*; program(s) used to solve structure: *SHELXS97* (Sheldrick, 2008 ▶); program(s) used to refine structure: *SHELXL97* (Sheldrick, 2008 ▶); molecular graphics: *X-SEED* (Barbour, 2001 ▶); software used to prepare material for publication: *publCIF* (Westrip, 2009 ▶).

## Supplementary Material

Crystal structure: contains datablocks global, I. DOI: 10.1107/S1600536809024623/ci2836sup1.cif

Structure factors: contains datablocks I. DOI: 10.1107/S1600536809024623/ci2836Isup2.hkl

Additional supplementary materials:  crystallographic information; 3D view; checkCIF report

## Figures and Tables

**Fig. 1.**
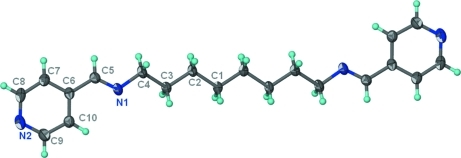
Displacement ellipsoid plot (Barbour, 2001) of C20H26N2 at the 70% probability level; H atoms are drawn as spheres of arbitrary radius.

## supplementary crystallographic information

### Experimental

1,8-Diaminooctane (0.145 g, 1 mmol) was dissolved in methanol (15 ml) and to this was added 4-pyridinecarboxaldehyde (0.215 g, 2 mmol). The mixture was heated for 4 h. The solid that formed was recrystallized from methanol in 70% yield; m.p. 393 K.

### Refinement

H atoms were placed in calculated positions (C-H = 0.95-0.99 Å) and were included in the refinement in the riding model approximation, with *U_iso_*(H) set to 1.2*U_eq_*(C).

### Crystal data

**Table:**

C_20_H_26_N_4_	*F*(000) = 348
*M**_r_* = 322.45	*D*_x_ = 1.192 Mg m^−^^3^
Monoclinic, *P*2_1_/*c*	Mo *K*α radiation, λ = 0.71073 Å
Hall symbol: -P 2ybc	Cell parameters from 1645 reflections
*a* = 11.6285 (4) Å	θ = 2.2–27.3°
*b* = 9.3821 (3) Å	µ = 0.07 mm^−^^1^
*c* = 8.8302 (3) Å	*T* = 140 K
β = 111.143 (2)°	Plate, light yellow
*V* = 898.52 (5) Å^3^	0.40 × 0.20 × 0.02 mm
*Z* = 2	

### Data collection

**Table:**

Bruker SMART APEX area-detector diffractometer	1588 reflections with *I* > 2σ(*I*)
Radiation source: fine-focus sealed tube	*R*_int_ = 0.023
graphite	θ_max_ = 27.5°, θ_min_ = 1.9°
ω scans	*h* = −15→15
6110 measured reflections	*k* = −12→11
2065 independent reflections	*l* = −11→11

### Refinement

**Table:**

Refinement on *F*^2^	Primary atom site location: structure-invariant direct methods
Least-squares matrix: full	Secondary atom site location: difference Fourier map
*R*[*F*^2^ > 2σ(*F*^2^)] = 0.048	Hydrogen site location: inferred from neighbouring sites
*wR*(*F*^2^) = 0.138	H-atom parameters constrained
*S* = 1.02	*w* = 1/[σ^2^(*F*_o_^2^) + (0.0697*P*)^2^ + 0.2588*P*] where *P* = (*F*_o_^2^ + 2*F*_c_^2^)/3
2065 reflections	(Δ/σ)_max_ = 0.001
109 parameters	Δρ_max_ = 0.31 e Å^−^^3^
0 restraints	Δρ_min_ = −0.23 e Å^−^^3^

### Fractional atomic coordinates and isotropic or equivalent isotropic displacement parameters (Å^2^)

**Table:**

	*x*	*y*	*z*	*U*_iso_*/*U*_eq_	
N1	0.28339 (13)	0.52618 (14)	0.97725 (16)	0.0353 (3)	
N2	0.09040 (12)	0.40434 (14)	1.39134 (15)	0.0326 (3)	
C1	0.48329 (13)	0.46639 (14)	0.56773 (16)	0.0248 (3)	
H1A	0.5601	0.4388	0.6573	0.030*	
H1B	0.4353	0.3784	0.5261	0.030*	
C2	0.40867 (13)	0.56437 (15)	0.63471 (17)	0.0264 (3)	
H2A	0.4584	0.6500	0.6818	0.032*	
H2B	0.3340	0.5961	0.5442	0.032*	
C3	0.36981 (13)	0.49380 (15)	0.76402 (17)	0.0265 (3)	
H3A	0.3086	0.4184	0.7125	0.032*	
H3B	0.4426	0.4476	0.8451	0.032*	
C4	0.31509 (18)	0.59651 (17)	0.8495 (2)	0.0413 (4)	
H4A	0.3748	0.6740	0.8979	0.050*	
H4B	0.2400	0.6397	0.7697	0.050*	
C5	0.20006 (13)	0.58108 (14)	1.01734 (16)	0.0250 (3)	
H5	0.1598	0.6647	0.9631	0.030*	
C6	0.16301 (12)	0.51912 (15)	1.14647 (15)	0.0225 (3)	
C7	0.07925 (13)	0.58973 (15)	1.19920 (17)	0.0265 (3)	
H7	0.0446	0.6782	1.1522	0.032*	
C8	0.04704 (13)	0.52910 (16)	1.32146 (18)	0.0303 (4)	
H8	−0.0092	0.5793	1.3576	0.036*	
C9	0.17031 (14)	0.33682 (16)	1.33808 (18)	0.0304 (3)	
H9	0.2018	0.2473	1.3852	0.036*	
C10	0.20944 (13)	0.38956 (15)	1.21893 (17)	0.0263 (3)	
H10	0.2673	0.3380	1.1869	0.032*	

### Atomic displacement parameters (Å^2^)

**Table:**

	*U*^11^	*U*^22^	*U*^33^	*U*^12^	*U*^13^	*U*^23^
N1	0.0532 (8)	0.0291 (7)	0.0383 (7)	0.0066 (6)	0.0343 (7)	0.0070 (6)
N2	0.0390 (7)	0.0354 (7)	0.0306 (7)	−0.0071 (5)	0.0212 (6)	−0.0029 (5)
C1	0.0282 (7)	0.0273 (7)	0.0236 (7)	0.0019 (5)	0.0148 (6)	0.0008 (5)
C2	0.0339 (7)	0.0257 (7)	0.0274 (7)	−0.0007 (6)	0.0203 (6)	0.0006 (6)
C3	0.0338 (7)	0.0256 (7)	0.0260 (7)	0.0022 (6)	0.0181 (6)	0.0023 (6)
C4	0.0682 (11)	0.0283 (8)	0.0490 (10)	0.0084 (7)	0.0473 (9)	0.0090 (7)
C5	0.0318 (7)	0.0225 (7)	0.0238 (7)	0.0000 (5)	0.0139 (6)	0.0003 (5)
C6	0.0244 (7)	0.0245 (7)	0.0202 (6)	−0.0037 (5)	0.0101 (5)	−0.0032 (5)
C7	0.0270 (7)	0.0277 (7)	0.0274 (7)	0.0002 (5)	0.0130 (6)	−0.0023 (6)
C8	0.0304 (7)	0.0348 (8)	0.0323 (8)	−0.0035 (6)	0.0191 (6)	−0.0066 (6)
C9	0.0387 (8)	0.0270 (7)	0.0297 (7)	−0.0027 (6)	0.0174 (6)	0.0010 (6)
C10	0.0308 (7)	0.0252 (7)	0.0267 (7)	0.0001 (5)	0.0151 (6)	−0.0017 (5)

### Geometric parameters (Å, °)

**Table:**

N1—C5	1.2558 (18)	C3—H3B	0.99
N1—C4	1.4643 (18)	C4—H4A	0.99
N2—C8	1.334 (2)	C4—H4B	0.99
N2—C9	1.3421 (18)	C5—C6	1.4758 (18)
C1—C1^i^	1.522 (2)	C5—H5	0.95
C1—C2	1.5225 (18)	C6—C10	1.3889 (19)
C1—H1A	0.99	C6—C7	1.3898 (18)
C1—H1B	0.99	C7—C8	1.3860 (19)
C2—C3	1.5227 (18)	C7—H7	0.95
C2—H2A	0.99	C8—H8	0.95
C2—H2B	0.99	C9—C10	1.3800 (19)
C3—C4	1.4998 (19)	C9—H9	0.95
C3—H3A	0.99	C10—H10	0.95
			
C5—N1—C4	117.82 (13)	C3—C4—H4A	109.3
C8—N2—C9	116.48 (12)	N1—C4—H4B	109.3
C1^i^—C1—C2	113.46 (14)	C3—C4—H4B	109.3
C1^i^—C1—H1A	108.9	H4A—C4—H4B	108.0
C2—C1—H1A	108.9	N1—C5—C6	121.75 (13)
C1^i^—C1—H1B	108.9	N1—C5—H5	119.1
C2—C1—H1B	108.9	C6—C5—H5	119.1
H1A—C1—H1B	107.7	C10—C6—C7	117.76 (12)
C3—C2—C1	113.18 (11)	C10—C6—C5	121.82 (12)
C3—C2—H2A	108.9	C7—C6—C5	120.42 (12)
C1—C2—H2A	108.9	C8—C7—C6	118.97 (13)
C3—C2—H2B	108.9	C8—C7—H7	120.5
C1—C2—H2B	108.9	C6—C7—H7	120.5
H2A—C2—H2B	107.8	N2—C8—C7	123.85 (13)
C4—C3—C2	113.11 (12)	N2—C8—H8	118.1
C4—C3—H3A	109.0	C7—C8—H8	118.1
C2—C3—H3A	109.0	N2—C9—C10	123.94 (14)
C4—C3—H3B	109.0	N2—C9—H9	118.0
C2—C3—H3B	109.0	C10—C9—H9	118.0
H3A—C3—H3B	107.8	C9—C10—C6	119.00 (13)
N1—C4—C3	111.63 (12)	C9—C10—H10	120.5
N1—C4—H4A	109.3	C6—C10—H10	120.5
			
C1^i^—C1—C2—C3	176.99 (14)	C5—C6—C7—C8	−179.57 (13)
C1—C2—C3—C4	170.37 (14)	C9—N2—C8—C7	0.5 (2)
C5—N1—C4—C3	−154.80 (15)	C6—C7—C8—N2	−1.1 (2)
C2—C3—C4—N1	−177.71 (14)	C8—N2—C9—C10	0.6 (2)
C4—N1—C5—C6	−179.09 (14)	N2—C9—C10—C6	−1.0 (2)
N1—C5—C6—C10	−6.5 (2)	C7—C6—C10—C9	0.4 (2)
N1—C5—C6—C7	173.65 (13)	C5—C6—C10—C9	−179.46 (13)
C10—C6—C7—C8	0.6 (2)		

Symmetry codes: (i) −*x*+1, −*y*+1, −*z*+1.
